# Is There a Chance of Normal Life in Severe Laryngeal web

**DOI:** 10.22038/IJORL.2021.55667.2924

**Published:** 2021-11

**Authors:** Raffaella Nenna, Greta Di Mattia, Laura Petrarca, Antonella Frassanito, Giancarlo Tancredi, Desiree Mollicone, Roberto Baggi, Fabio Midulla

**Affiliations:** 1 *Department of Maternal Infantile and Urological Sciences, Sapienza University of Rome, Rome, Italy.*; 2 *Respiratory Endoscopy Unit, Department of Pediatric Anesthesia and Intensive Care, Meyer Children Hospital, Florence, Italy.*

**Keywords:** Airway, Stenosis/reconstruction, Larynx, Pediatric airway

## Abstract

**Introduction:**

Laryngeal web is a rare cause of pediatric stridor and respiratory distress. The clinical presentation is variable and symptoms usually correlate with the severity of the airway obstruction.

**Case Reports:**

We describe the cases of three children unexpectedly diagnosed with laryngeal web after a severe episode of bronchiolitis and after thirteen and eleven years, respectively, of persistent symptoms despite asthma medications.

**Conclusion:**

Even if it is a rare cause of stridor and respiratory distress, congenital subglottic web could be a life threatening condition and clinicians should always consider it in the differential diagnosis of persistent noisy breathing, even in adolescents and young adults.

## Introduction

Laryngeal web is a rare condition characterized by the presence of abnormal tissue between two parts of the larynx (1). It is mostly acquired and usually develops after intralaryngeal surgery or traumatic intubation. Congenital webs are less common, representing only 5% of congenital laryngeal anomalies, and they derive from an incomplete recanalization of the larynx during embryogenesis (2). Among congenital webs, the most common is the anterior glottic type, with or without subglottic extension, while isolated subglottic webs are extremely rare (3). The clinical presentation is variable and could be misleading, but most of the children have respiratory distress, dysphonia, abnormal cry, hoarseness or stridor (1,4). We described the cases of three congenital laryngeal webs, belatedly and eventually diagnosed because of insidious symptoms.

## Case Reports

Case 1 

Case 1 is a 35-day-old girl born to unrelated healthy Caucasian parents. Pregnancy, delivery at term and postnatal period were uneventful, Apgar scores were 9 at 1 minute and 10 at 5 minutes and birth weight was 3250 g. At 14 days of life, due to the development of severe respiratory distress with cyanosis, the patient was admitted to the Pediatric Intensive Care Unit of our hospital with a diagnosis of respiratory syncytial virus (RSV) bronchiolitis. She required oxygen supplementation by orotracheal intubation for 5 days and, after 13 days, was discharged in good conditions. However, 4 days later, she was readmitted to the Pediatric Emergency Room with cough and mild stridor. 

**Fig 1:**
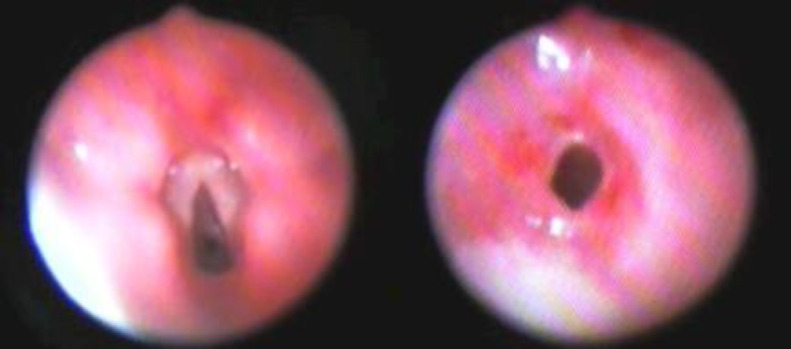
Flexible fibrobronchoscopy (Pentax 3.5 mm) revealed an isolated laryngeal web

At physical examination, the child had respiratory distress, with jugular retractions, and parasternal inspiratory monophonic wheezing. For this reason, a fibrobronchoscopy (Pentax 3.5 mm) was performed and an isolated membranous subglottic web was diagnosed and subsequently removed with laser lysis (Fig.1). 

Case 2

Case 2 is a 13-year-old boy born to unrelated healthy Caucasian parents. He was delivered by cesarean section at 38 gestational weeks. The Apgar scores were 9 at 1 minute and 9 at 5 minutes and birth weight was 3540 g. Approximately one hour after birth, he developed “noisy breathing” and increasing respiratory distress, which led to orotracheal intubation for 7 days; right after the intubation and on day 5, blood was suctioned from the tube. After 21 days, the patient was discharged in good condition with a diagnosis of *Pseudomonas *pneumonia. Parents reported wellness until he was 2-year-old, when he developed an expiratory "breath sound" that increased with exercise. 

For this reason, he had been followed for asthma by a Pediatric Pulmonologist until the age of 6 years, but, despite asthma therapy, symptoms did not improve. At the age of 12 years, the patient performed a spirometry to receive a certificate of fitness for sport, but despite the abnormal shape of the flow-volume curve, the spirometry was considered normal and he started playing basketball.

**Fig 2:**
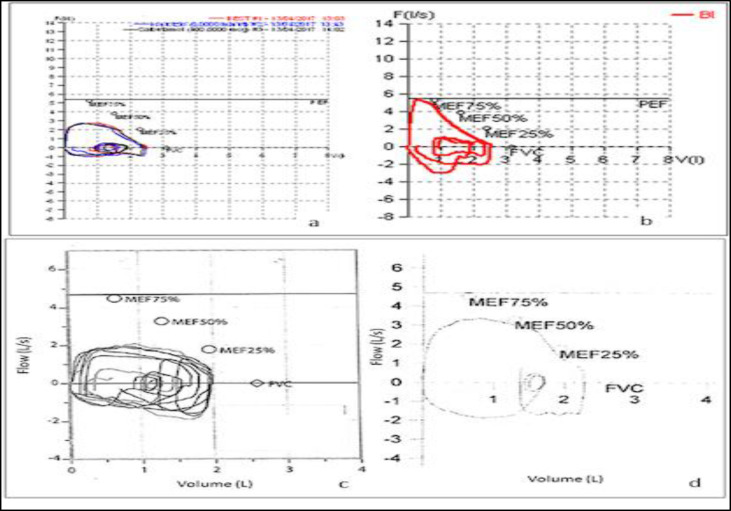
Case 2 (a and b) and Case 3 (c and d), spirometries. The patients performed a spirometry (a: before stress test, after stress test and after salbutamol 400 mcg, c: before and after salbutamol 400 mcg) which revealed a extra thoracic obstruction. One month (b) and three months (d) after the web removal, the spirometries were normal

After 3 months, to investigate a sinus arrhythmia, he underwent an exercise stress test (Bruce test); at rest, vital parameters were as follow: heart rate (HR) 75 beats per minute (bpm), respiratory rate (RR) 26 breaths per minute and peripheral oxygen saturation (SpO_2_) 96% at room air. His weight was 45 kg (40° centile) and his height 154 cm (29° centile). Before the stress test, after it and after administration of bronchodilator (salbutamol 400 mcg) three spirometries were performed (Fig.2a). 

Insofar as the spirometries were suggestive for an extra thoracic obstruction and the respiratory sound was audible during the test, a bronchoscopy was requested. During flexible fibrobronchoscopy (Pentax 3.5 mm), a fibrotic subglottic web, obstructing almost completely the airway, was found.

**Fig 3:**
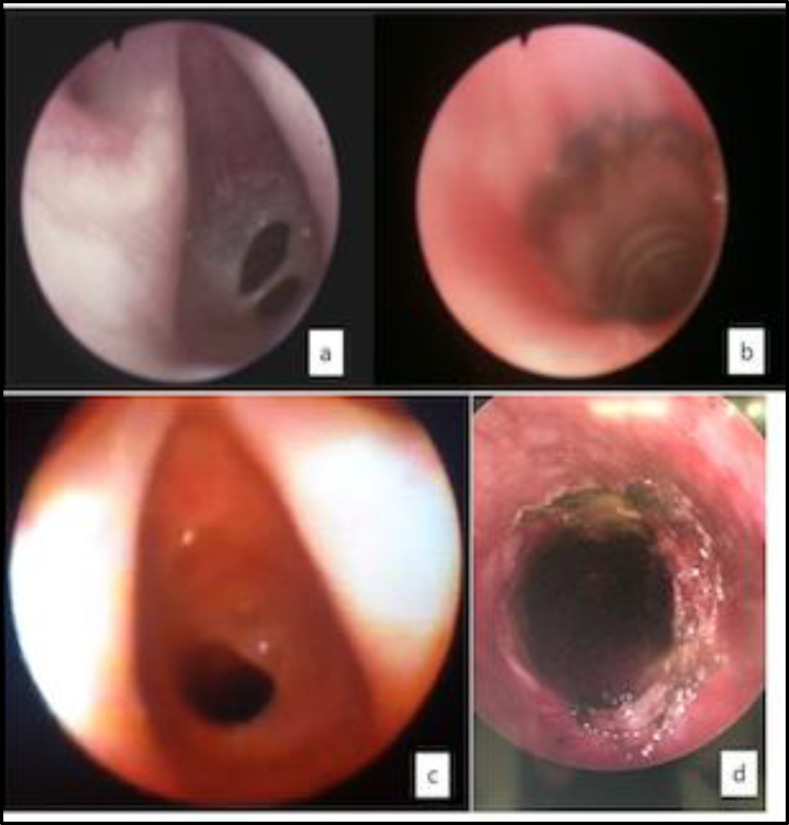
diagnostic and therapeutic videobroncho- scopy. Flexible videobronchoscopy (Karl Storz Germany 5.2 mm) revealed a fibrotic subglottic web (a and c), which was removed with cold instruments (Potts scissors) and diode laser (length wave 980 nm, power 5 Watts) (b and d)

The web was further evaluated with videobronchoscopy (Karl Storz Germany 5.2 mm) (Fig.3a), removed with cold instruments (Potts scissors) and diode laser (length wave 980 nm, power 5 Watts) and larynx was dilated with balloon calibrated at 10-11-12 mm (Fig. 3b). 

The day after, the boy was discharged in good condition. One month later we repeated a spirometry and a flexible videobronchoscopy (Karl Storz Germany 5.2mm) (Fig.2b), which were completely normal. His parameters were as follow: HR 75 bpm, RR 18 breaths per minute and SpO_2_ 99% at room air. 

Case 3

Case 3 is an 11-year-old boy born to unrelated healthy Caucasian parents. He was delivered by cesarean section at 38+2 gestational weeks for obstructed labor. The Apgar scores were 9 at 1 minute and 10 at 5 minutes and birth weight was 3380 g. Due to tachypnea and increasing respiratory distress he was transferred to the pediatric intensive care unit and intubated for 7 days with sudden improvement of the respiratory pattern. 

After 19 days, the patient was discharged in good condition. Parents reported occasional episodes of respiratory distress that increased with exercise (football and swimming). For this reason, he had been followed for asthma by a Pediatric Pulmonologist for 4 years, without improvement. At the age of 6 years, because of a bicuspid aortic valve the patient underwent an exercise stress test (Bruce test) that was normal. At the age of 11 years the child underwent a spirometry that showed a forced expiratory volume in 1 second (FEV_1_): 1.78 l (79%), forced vital capacity (FVC): 2 l (78%), FEV_1_/FVC: 88.8 % and peak expiratory flow (PEF): 2.1 l/s (45%) with no response to inhaled bronchodilator (salbutamol 400 mcg). 

Due to the typical extra-thoracic obstruction of the spirometry (Fig. 2c), the patients underwent a flexible bronchoscopy (Pentax 3.5 mm) that showed a fibrotic subglottic web, obstructing 2/3 of the airway. The web was removed with cold instruments (Potts scissors) and diode laser (length wave 980 nm, power 5 Watts) (Fig. 3d) with sudden improvement of symptoms. 

Three months a marked improvement of spirometric (Fig.2d) and flexible videobronchoscopic (Karl Storz Germany 5.2mm) evaluations were demonstrated with good voice quality.

## Discussion

Congenital incomplete laryngeal webs have variable clinical manifestations, but they usually become symptomatic during the first months of life, presenting with stridor or respiratory distress (1). If the diagnosis is not promptly made and the patient develops respiratory distress, intubation is generally required and it is hypothesized that many pure congenital webs are mechanically broken during blind emergency endotracheal intubation. For this reason, they are often underdiagnosed (3). 

Something similar happened to our patients: case 1 developed severe respiratory distress 14 days after birth, triggered by an RSV infection, and case 2 and 3 immediately after birth and, in all cases, intubation was required and probably created a lesion to the congenital web. In particular, in case 2, blood came out from the tube right after the intubation and 5 days later, suggesting that some structures of the airway were damaged. Moreover, even if we cannot exclude that the webs were secondary to intubation, we considered them congenital because of their fibrotic structure and the absence of subglottic stenosis at the endoscopic evaluation (Figure 1, 3a and 3c). 

The symptoms of our patients were misdiagnosed and a diagnosis of laryngeal web was not promptly made. In case 1, the respiratory distress was thought to be a consequence of RSV bronchiolitis and a congenital malformation was not considered until the child was 35 days old. In case 2 and 3, symptoms were misdiagnosed as asthma for many years, although they did not improve with bronchodilators and anti-inflammatory drugs. This is not the only case of a misdiagnosed laryngeal web described in literature: Linna et al described 14 cases of central airway stenosis misdiagnosed as asthma and one of them was a laryngeal web (5). 

However, when a suspected asthmatic patient does not improve with different medications, it is mandatory to perform further examination, such as bronchoscopy. Unfortunately, our patient 2 was not correctly evaluated even at 12 years, despite the flow-volume curve was highly suggestive for an upper respiratory tract obstruction that was interpreted as poor compliance. Even the type of “noisy breathing” is difficult to categorize. Parents of patients 2 and 3 reported a sort of recurrent stridor that was often audible and mainly exacerbated by exercise. The most frequent congenital condition associated with stridor is airway malacia but sometimes other anomalies should be considered such as laryngeal cysts or webs (6,7). Bronchoscopy is the gold standard to evaluate laryngeal malformations: it provides anatomical and functional information of the larynx and can directly assess the mucosa and the presence of inflammation (8,9). In our patients, webs were removed with laser, which is the best option when subglottic stenosis is not associated, because it cuts precisely and causes minimal damage and bleeding to the surrounding laryngeal structures (3,8). 

It is interesting to understand how case 2 lived for 13 years with such severe malformation, with few symptoms. 

The mother described the child's respiratory sound as a noisy breathing associated with mild shortness of breath during exercise, not severe enough to stop him. He grew up well (weight: 40° centile, height: 29° centile) and has always been healthy. He was able to play basketball 5 hours per week, the flute and do theatre with few complaints. He probably compensated his low tidal volume with a higher RR (26 breaths per minute), which returned to normal values (18 breaths per minute) one month after the web removal. 

## Conclusions

The presented cases have been initially misdiagnosed, and patient tolerated it for long. Congenital subglottic web is a rare cause of stridor and respiratory distress. However, this condition could be life threatening and clinicians should always consider it in the differential diagnosis of persistent noisy breathing, even in adolescents and young adults. 
